# Artificial Intelligence and Digital Biomarkers for Early Detection and Monitoring of Neurological Disorders: A Narrative Review

**DOI:** 10.3390/diagnostics16172799

**Published:** 2026-08-31

**Authors:** Arshad Husain Rahmani, Tarique Sarwar

**Affiliations:** Department of Medical Laboratories, College of Applied Medical Sciences, Qassim University, Buraydah 51452, Saudi Arabia

**Keywords:** machine learning, artificial intelligence, neurological disorders, digital biomarkers

## Abstract

Neurological disorders like Alzheimer’s disease, Parkinson’s disease, and epilepsy are becoming major causes of disability and mortality worldwide, and their prevalence is expected to rapidly increase with the aging of the population. These diseases develop silently, with irreversible neuronal damage often occurring decades before any clinical signs of illness are noticed, making early diagnosis and treatment difficult. The presymptomatic period greatly restricts the effectiveness of therapeutic interventions and reduces the possibility of disease-modifying interventions. Traditional diagnostic methods based on clinical assessment, neuroimaging, and invasive biomarkers are not sensitive enough to identify the disease at an early stage and are expensive to the healthcare system. The latest artificial intelligence (AI) technology and machine learning (ML) approaches, together with digital biomarkers obtained from eye tracking, facial expressions, speech analysis, motor dynamics, electrophysiology, wearable devices, and passive sensing, offer promising non-invasive alternatives for early detection of diseases. However, most reported performance metrics are derived from retrospective or pre-validated datasets, and prospective external validation remains limited. This narrative review synthesizes current evidence on AI-driven digital biomarkers for early detection of neurological diseases, examining disease-specific applications, methodological approaches, and challenges in clinical practices. We emphasize that clinical utility is task specific and dependent on disease stage, validation design, clinical endpoints, cost, workflow integration, and availability of disease-modifying therapies. We also note that much of the evidence summarized here derives from retrospective, case-control, or internally validated datasets and that prospective, patient-independent, and external validation with clinically meaningful endpoints remains limited. Reported performance figures should be read as proof-of-concept evidence rather than as evidence of demonstrated clinical readiness. We highlight promising future directions, including federated learning, explainable AI, and precision neurology approaches, while acknowledging that most applications remain investigational and require prospective validation before broad clinical deployment.

## 1. Introduction

Neurological disorders such as Alzheimer’s disease (AD) and Parkinson’s disease (PD), as well as epilepsy, represent a significant global health issue, as they are among the leading causes of disability and mortality worldwide [1,2,3]. These disorders often progress with asymptomatic onset during the prodromal or initial stages, which leads to considerable neuronal damage, such as 30–70% loss of substantia nigra dopaminergic neurons in PD, before motor or cognitive symptoms are manifested. This limits therapeutic opportunities and prevents disease-modifying treatments [1,2,4]. In the absence of curative therapies and amid projections of exponential prevalence increases attributable to aging populations, early detection is essential to facilitate timely management, personalize care strategies, and mitigate socioeconomic impacts [1,3,5].

Traditional diagnostics, which are based on subjective clinical examination, neuroimaging, symptom-based tests, and bioindicators including DAT-SPECT or CSF analysis, have poor sensitivity at early stages of the disease and are resource intensive, and confirmation is delayed after severe pathology [3,4,6,7]. In contrast, multimodal data are used to achieve accurate early detection with artificial intelligence (AI) and machine-learning (ML) paradigms. These paradigms use digital biomarkers obtained from remote-sensing modalities, such as eye tracking, speech and voice analysis, wearables, facial expression, EEG, and motor dynamics, and harness multimodal data for precise, non-invasive early identification [1,6,8,9,10]. Recent studies demonstrate the potential of AI-enabled digital biomarkers across multiple neurological disorders. For example, convolutional neural networks trained on eye-tracking data achieve ROC-AUC values of up to 0.88 to differentiate PD [1]. Speech-based natural language processing (NLP) systems can predict the transition from mild cognitive impairment (MCI) to AD progression with 78.5% accuracy and 81.1% sensitivity [9]. A hybrid CNN-BiLSTM model with attention mechanism achieved an AUC of 0.91 for the detection of PD-related FoG using wearable sensors [11]. Deep learning approaches have achieved high performance in benchmark EEG datasets for seizure detection [6,12]. Wearable AI detects sleep apnea (a prodromal correlate) with a pooled accuracy of 0.893 [8]. However, these performance estimates originate predominantly from retrospective or internally validated datasets and should not be interpreted as equivalent to real-world clinical performance. Prospective, patient-independent, multicenter validation remains limited, and clinical utility should therefore be interpreted according to the intended clinical application and validation design.

Bibliometric methods demonstrate that AI-neuroscience research has drastically risen since the mid-2010s, with multimodal fusion, deep-learning methods, and applications that target the PD prodrome using ECG, MRI, and AI models reaching sensitivity levels of up to 84% [2,4,5]. These technologies are transforming clinical paradigms into precision neurology, ultimately improving the areas of prevention, risk stratification, and clinician decision making [3,10]. This narrative review outlines AI/ML methods; performance in AD, PD, and epilepsy; integration issues; ethics; and future directions, such as federated learning and explainable AI. Extensive systematic reviews have revealed important utility for ML approaches (including deep learning) in neurodegenerative and neurocognitive diseases, with twelve and eighteen algorithms identified as effective for detecting dementia and Parkinson’s disease, respectively [13]. At the same time, there are other emerging models, such as transformer-based architectures and graph neural networks that are also being developed as effective tools for the detection of epilepsy using EEG, and these tools are able to model complex spatiotemporal relationships in neural signals [14,15]. This review is a narrative rather than a systematic review; its literature search and selection approach is described in Section 2. For clarity, we define artificial intelligence (AI) as the broad field concerned with building systems that perform tasks normally requiring human intelligence, machine learning (ML) as a major subset of AI in which algorithms learn patterns from data rather than following explicit rules, and deep learning (DL) as a specialized subset of ML using multilayer neural networks capable of learning hierarchical representations directly from raw or minimally processed data. These terms are not interchangeable and are used precisely throughout this review. We define a digital biomarker as a characteristic derived from a digitally acquired behavioral, physiological, or sensor-based signal (for example, speech, eye movements, gait, EEG, or wearable sensor output) that is objectively measured and used as an indicator of a biological or disease-related process. This is distinct from digitized conventional biomarkers (for example, MRI, PET, CSF, or genomic measures), which we discuss only where they are combined with digital biomarkers in multimodal frameworks. We also distinguish and use consistently six clinical tasks that are frequently conflated in the literature as “early detection”: (i) population screening of asymptomatic individuals; (ii) diagnostic classification of clinically established disease; (iii) differential diagnosis; (iv) prognostic or conversion prediction (for example, MCI to dementia or prodromal to manifest disease); (v) longitudinal disease or symptom monitoring; and (vi) acute event forecasting (for example, seizure prediction). Throughout this review, we specify, wherever primary literature allows which of these tasks a given study actually addresses since strong discrimination between an established disease group and healthy controls does not, by itself, establish capability for presymptomatic or prodromal screening.

## 2. Methods

This is a narrative review of AI and digital biomarker applications in early detection of neurological disorders. The review was conducted through searches of PubMed/MEDLINE, Scopus, Web of Science, and IEEE Xplore with a focus on publications from 2015 to 2025 using the terms “artificial intelligence”, “machine learning”, “deep learning”, and “digital biomarker” combined with disease terms (“Alzheimer’s disease”, “Parkinson’s disease”, “epilepsy”) and modality terms (“eye-tracking”, “speech analysis”, “wearable sensors”, “facial expression analysis”, “EEG”, “gait analysis”). We included peer-reviewed studies, and where relevant, preprints and conference proceedings, reporting the development or validation of AI or ML based digital biomarkers for the screening, diagnosis, differential diagnosis, prognosis, or longitudinal monitoring of Alzheimer’s disease, Parkinson’s disease, or epilepsy in human participants published in English. We excluded studies focused exclusively on conventional neuroimaging or fluid biomarkers without a digital biomarker component, animal studies, and non-peer-reviewed sources other than the preprints noted above.

This review focuses on Alzheimer’s disease, Parkinson’s disease, and epilepsy as exemplar neurological disorders with the largest and most methodologically mature digital-biomarker literatures; we use “neurological disorders”, rather than “neurodegenerative diseases”, in the title and throughout because epilepsy is not itself a neurodegenerative condition.

## 3. Global Burden of Neurological Disorders

Neurological disorders are the primary cause of combined disability and mortality on the global scale. The 2021 Global Burden of Disease tudy attributes 443 million disability-adjusted life years (DALYs) to disorders of the nervous system, representing an 18% increase since 1990 and affecting an estimated 3.4 billion people (43% of the global population) [3,16]. This burden is expected to increase with increasing age, lifestyle trends, pollution, climate change, and the post-COVID conditions [3]. The burden of diseases falls on low- and middle-income countries [16]. Dementia, for which AD is the most common cause (accounting for an estimated 60–70% of cases), is currently estimated to affect approximately 55 million people worldwide (WHO, 2023), a figure projected to increase to 139 million by 2050 [1,17]. PD is the second most prevalent neurodegenerative disease, affecting an estimated 11.8 million people worldwide in 2021 and projected to reach approximately 25.2 million by 2050. This corresponds to roughly 3% of people above 65 and up to 5% above 85 years old, with escalating economic costs from medical expenses and lost productivity [1,2,18]. Epilepsy affects nearly 50 million people around the world, and seizures still cannot be reliably predicted in terms of when, how powerful, or how long they will last [12]. The global prevalence, projected growth, and disability burden of major neurological disorders are summarized in Table 1 [3,16,17,18].

These disorders progress with substantial neuronal loss, such as 30–70% in the substantia nigra of PD or 50% of dopaminergic neurons loss, which occurs years or decades before motor, cognitive, or symptomatic manifestations emerge, significantly reducing therapeutic opportunities given that there are no curative treatments [1,2,4]. Early diagnosis is therefore essential to facilitate neuroprotective interventions, disease-modifying treatments, and individualized treatment, while also alleviating the socioeconomic burden. This need is highlighted by the WHO Intersectoral Global Action Plan on Epilepsy and Other Neurological Disorders 2022–2031, which prioritizes brain health and global collaboration [3].

## 4. Role of Artificial Intelligence and Machine Learning in Diagnosis

AI and ML paradigms differ significantly in their value for diagnosis across architecture, modality, and disease stage. In the low-data regime, conventional SVMs and gradient-boosted ensembles offer advantages compared to supervised deep learning. In the high-data regime, they offer superior calibration for clinical risk scores, with training data exceeding ~500 annotated cases [19,20]. Importantly, early or intermediate fusion schemes (MRI, PET and CSF, or multimodal wearable fusion [accelerometer, gyroscope, cardiac signals]) consistently result in greater AUC gains over unimodal analysis (gait, CSF, or PET) for the classification of AD (8–12% for the former [21,22] and 0.07–0.14% for the latter [4,11] when applied to PD patients). This performance gap illustrates that fusion design and architectural choices materially influence discriminative performance; however, as discussed in Section 6.2, translational viability ultimately depends on the interaction among modality availability, cost, invasiveness, missing data robustness, and clinical workflow constraints, and not on architecture alone [23]. In contrast to traditional diagnostics, which are limited by subjectivity and limited pre-symptomatic sensitivity, deep learning architectures, such as convolutional neural networks (CNNs) and recurrent neural networks, allow the recognition of patterns automatically, with better performance. CNNs on eye-tracking data provide an ROC-AUC of up to 0.88 for PD [1]; speech-based natural language processing (NLP) models are able to predict the progression of MCI to AD with 78.5% accuracy and 81.1% sensitivity [9]. ML on EEG advances epilepsy seizure prediction [6]. A 2025 systematic review of 23 real-time EEG seizure-prediction studies reports a deep learning peak accuracy of 99.01% and sensitivity of 99.81% [12]. ECG-AI models reach 84% sensitivity for prodromal PD [2,4].

According to bibliometric trends, AI in neuroscience research has demonstrated exponential growth since the 2010s, with supervised and unsupervised learning, granular computing to cognitive phenotyping, and predictive learning to risk stratification [1,5]. This shift supports precision neurology, promoting prognostic accuracy, decision support for clinicians, and scalable screening in resource-constrained environments with privacy-preserving techniques [3,4,10,24]. Moreover, multimodal AI models that combine genomics, clinical imaging, and electronic health record (EHR) data are creating truly personalized ideas for neurological disease management [25].

## 5. Therapeutic Windows and Disease Modification Strategies

The ability to detect people at the early stages of neurodegenerative disease, before a significant number of neurons have been lost or subjected to irreversible pathological changes, is crucial for the timely use of disease-modifying interventions that could slow, halt, or prevent disease progression [26]. Molecular biomarkers, such as neurofilament light chain, along with the high-tech liquid biopsy modalities of exosomal tau and α-synuclein, play a fundamental role in non-invasive diagnostic approaches and show a future opportunity in the early diagnosis of the disease before its clinical presentation occurs, that is, before overt clinical symptoms emerge [27,28]. However, despite these technological improvements, current standard diagnostic methods, such as PET imaging and lumbar puncture, remain invasive, expensive, and resource intensive, limiting their wider use, especially for early-stage disease screening [29].

### 5.1. AI-Driven Digital Biomarkers

AI based digital biomarkers that are based ML algorithms applied to data collected from wearables, sensors, speech, eye-tracking devices, gait detectors, and EEG offer non-invasive, objective, presymptomatic detection of neurodegenerative diseases such as AD, PD, and epilepsy, thus overcoming the limitations of traditional diagnostics [1,6,10]. High-frequency phenotypic and multimodal AI approaches are proving powerful for early detection of neurology diseases. Techniques like granular computing rules on BIOCARD data, CNN-based eye tracking (ROC-AUC 0.88), and NLP processing tools (accuracy 78.5%, sensitivity 81.1%) achieve high sensitivity and specificity for early detection [1,9,24]. Similarly, multimodal AI fusion shows high performance with prodromal Parkinsonism, where ECG models achieve 84% sensitivity, neuromelanin images achieve 95% specificity, and accelerometry demonstrates better performance than the genetic or lifestyle data [4]. Deep learning models using wearable and gait-based data such as convolutional and recurrent neural networks can automatically extract features from heterogeneous sources with more than 90% accuracy for PD [2,11]. Digital phenotyping in this paradigm shift contributes to scalable screening, personalized interventions, and enrichment of clinical trial populations [1,9,24]. The major categories of AI-enabled digital biomarkers, associated data features, and representative neurological applications are outlined in Table 2. Representative AI-enabled digital biomarker studies are presented according to their intended clinical application, disease stage, digital biomarker modality, validation strategy, and reported diagnostic performance. Because the included studies differ substantially in patient populations, datasets, reference standards, validation methods, and intended clinical use, direct comparison of reported performance metrics across studies should be interpreted with caution. Where primary studies did not consistently report cohort size, external validation, or comparator models, these fields are indicated accordingly and are summarized in Table 3.

#### 5.1.1. Oculomotor Signatures and Eye Tracking

Oculomotor signatures derived from eye-tracking technologies provide non-invasive digital biomarkers of neurodegenerative disorders such as PD and AD, Eye-tracking approaches can detect subtle abnormalities in saccades, smooth pursuit, fixations, pupil behavior, blinking, and gaze patterns, providing potentially useful digital measures for early disease detection and characterization [1,13]. Such minor abnormalities can be detected using a video-based eye tracking systems and other technology-assisted approaches, supporting machine learning classification of prodromal and early-stage conditions [1,13]. In PD, video-based monitoring of saccades, pupil size, and blink frequency during pro/anti-saccade tasks has a sensitivity of 83%, specificity of 78%, and an ROC-AUC of 0.88, Data-driven analysis incorporating rapid eye movement measures and other clini-cal/biological features has demonstrated promising performance for early PD detection, with reported accuracy of 96%, sensitivity of 97%, and specificity of 95%. Fixation and saccade measures in neurodegenerative disorders are differentiated with 81% accuracy using convolutional neural networks trained on infrared oculography [1]. Eye-tracking visual inference tasks in AD and mild cognitive impairment are significant for discriminating between groups, as shown using linear mixed models [1]. The increasing availability of low-cost and remotely deployable digital technologies may facilitate the development of accessible screening approaches; however, further validation in larger, clinically characterized cohorts is required before eye-tracking-based biomarkers can be routinely incorporated into clinical practice [1,13].

#### 5.1.2. Speech and Voice Analysis

Neurodegenerative diseases are detected with speech and voice analysis based on remote recordings, smartphone applications, and neuropsychological interviews, as well as early disruption of prosody, phonation, speech fluency, rhythm, vocabulary use, linguistic patterns, and acoustic characteristics of dysphonia or dysarthria [4,9,24,30]. These non-obvious irregularities can be identified with automated transcription and processing using natural language processing (NLP), language models, convolutional neural networks (CNNs), or support vector machines (SVMs), enable machine learning-based prediction of prodromal and early disease. These methods are often more effective than traditional measurements [4,9,24,30]. In AD, transformer-based language models trained on Framingham Heart Study transcripts can predict the progression of MCI-to-AD within 6 years (78.5% accuracy and 81.1% sensitivity). By combining speech-based textual characteristics with demographic data, this model enables remote screening for cognitive impairment [9]. In PD, AI voice sample analysis of patients with isolated REM behavior disorder identifies high-level prosodic, pause, and rhythm features with balanced accuracies of 89% and 70% in males and females, respectively, and 63–70% in prodromal isolated REM behavior disorder. CNNs models have 90.45% accuracy on 10-s segments [4,30]. These tools can be used in longitudinal monitoring to scale up cost-effective presymptomatic detection in varied cohorts [8,25]. The combination of SHAP values with voice biomarkers has also been shown to achieve high accuracy (91.11%) and recall (92.50%) in early PD detection, providing clinical interpretability of acoustic features [29].

#### 5.1.3. Facial Expressions and Motor Dynamics

Facial expressions and motor dynamics derived from video recordings, smartphone apps, webcams, and remote testing identify early disruptions in hypomimia, facial bradykinesia, action units, emotional expressivity, saccadic movements, and overall facial mobility, thus providing non-invasive digital biomarkers of neurodegenerative diseases, such as PD [1,32]. These minor abnormalities, detected with automated systems like OpenFace that use Facial Action Coding System (FACS) to quantify the presence and intensity of action units, allow ML-based detection of prodromal and early stages. These systems are more accurate than traditional clinician-scored systems like the MDS-UPDRS item 3.2, which is used to measure facial expression [1,40,41,42]. In PD, hypomimia is supported by video clips of emotional expressions, such as happiness, disgust, and anger, showing much lower movement ranges in patients than in controls. Deep learning models achieve 85% accuracy in masked face detection, and statistical shape models capture day-to-day changes in symptoms [1,38,39].

Other motor dynamics such as gait and posture also can be analyzed with a view to increasing diagnostic potential. For example, smartphone accelerometer recordings and gyroscopes can be assessed using AI with a high level of accuracy to measure bradykinesia and gait disturbance in PD, providing an adjunct to traditional clinical measures that are more sensitive and more readily available [10,30,37]. Additionally, studies using wearable accelerometer data have shown that prodromal PD can be detectable several years prior to clinical diagnosis. This approach is more accurate, sensitive, and specific for identifying at-risk individuals than genetic and lifestyle models for distinguishing at-risk individuals from the general population [4]. Wearable sensor-based gait analysis has shown gait biomarkers (AUC 0.7–0.9) capable of differentiating PD subtypes from healthy controls and measuring disease severity longitudinally [43,44]. Data from the PADS smartwatch have been validated for classification of PD from various differential diagnoses, further supporting the clinical promise of multi-sensor wearable technology [45]. Importantly, such remote solutions are low cost and enable presymptomatic screening as well as longitudinal tracking across cohorts [1,46,47].

### 5.2. Cognitive and Behavioral Digital Phenotyping

Cognitive and behavioral digital phenotyping based on web-based cognitive tests, mobile applications, smartphone interactions, neuropsychological tests, and daily life technology enables the identification of early impairments in memory, executive function, reaction time, daily routines, and cognitive motor independence. These features serve as non-invasive digital biomarkers of neurodegenerative diseases such as AD and PD [1,24,33,48]. Minor abnormalities can be detected using granular computing rules, multi-granular AI models, rough set rules, and ML classifiers applied to datasets such as BIOCARD, allowing early classification of pre-dementia, MCI, and prodromal stages, often revealing impairments that do not show up with traditional neuropsychological testing [1,24,49].

It is important to distinguish clinically defined mild cognitive impairment (MCI) from biologically defined Alzheimer’s disease. MCI is a heterogeneous syndrome that may result from Alzheimer’s disease as well as vascular, Lewy body, frontotemporal, or other neurological disorders. Consequently, digital biomarkers that identify MCI or predict progression from MCI to dementia should not automatically be interpreted as detecting underlying Alzheimer’s disease pathology. Instead, AI-enabled digital biomarkers are most appropriately viewed as complementary tools that may identify individuals requiring confirmatory biological assessment using plasma biomarkers (e.g., p-tau217, GFAP, NfL), cerebrospinal fluid biomarkers, or amyloid PET imaging.

In AD, granular computing rules applied to BIOCARD cognitive data predict pre-dementia stages in normal subjects. Rough set rules using clinical dementia rating (CDR) sum of boxes detect early changes. Wed-based testing (cCOG) employing wordlist recall, reaction tasks, and trail-making tasks achieves ROC-AUC values 0.71–0.84 for MCI and 0.86–0.94 for dementia. The Altoida app demonstrates an AUC of 0.92 in predicting MCI-to-dementia transition [1,42,50]. In PD, intelligent granular computing shows cognitive change independent of motor symptoms, including differences between med-off/on states [1].

Low-cost remote platforms like Altoida and SASG support scalable presymptomatic screening, personalized monitoring, and clinical trial enrichment across cohorts [1]. These methods are especially easy to combine with computer vision AI models and voice analysis, which have proven highly diagnostic in predicting cognitive function based on passive data, including facial expressions, movements, and speech features [51]. Such multimodal integration allows the development of general digital phenotypes, indicating the multifactorial nature of neurodegenerative disease progression [1,52]. Importantly, predictive models derived from these data can identify individuals at high risk of amyloid deposition long before clinical onset using non-invasive measurements of lifestyle factors or APOE status, thereby reducing reliance on neuroimaging or blood-based biomarkers [53].

### 5.3. Passive Sensing from Wearables and Smartphones

**Figure 1:**
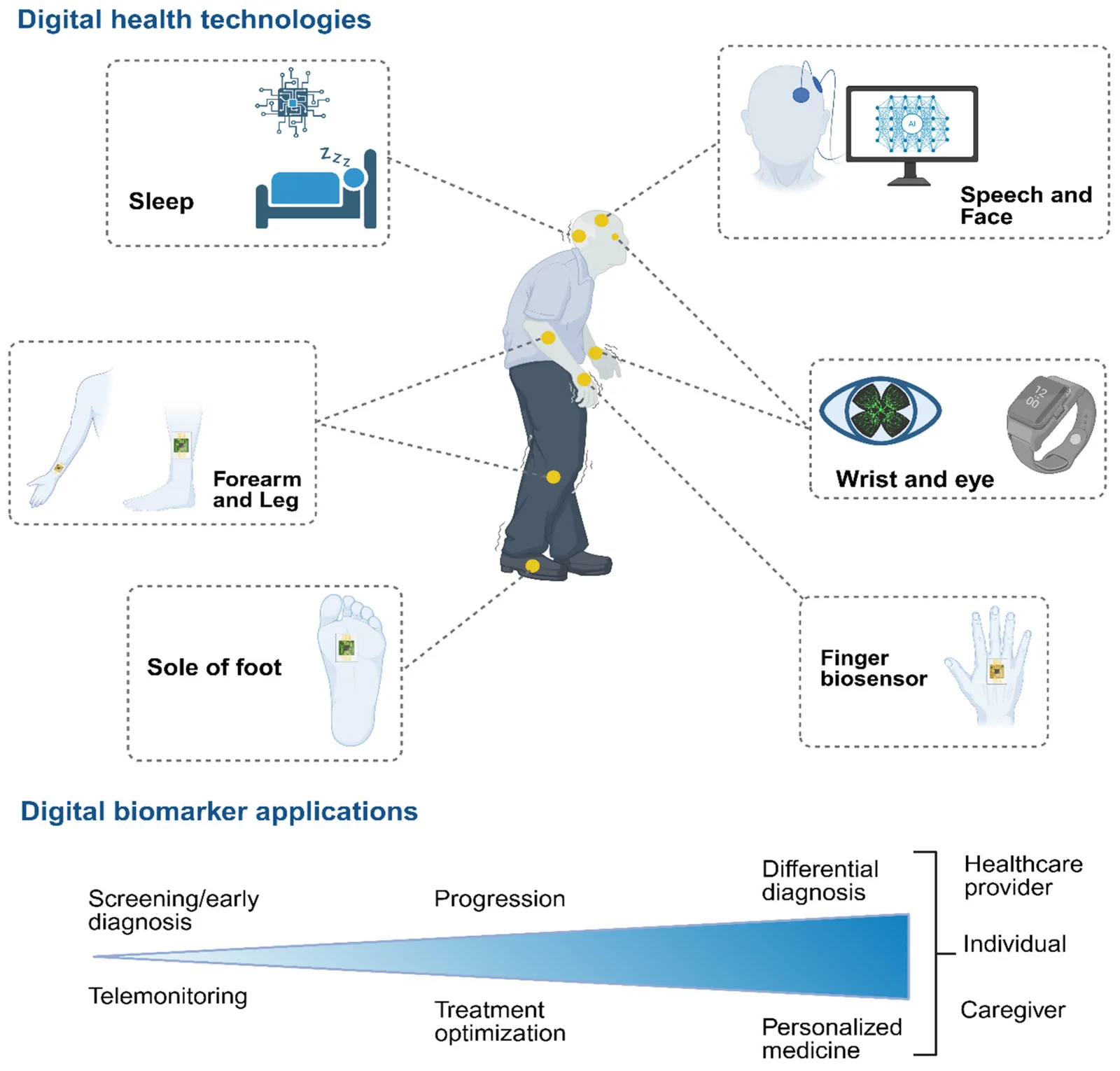
Multimodal digital biomarkers for neurological disease detection. Digital biomarkers are captured from speech, sleep, eye movements, facial expressions, finger interactions, wearable sensors (wrist and forearm), instrumented insoles, and lower limb monitoring systems. These multimodal data streams provide objective measures of cognitive, motor, behavioral, and physiological function for AI-driven early detection and monitoring of neurodegenerative disorders. The figure was created in https://BioRender.com.

Smartphones, wearable devices, and smart home systems allow data to be collected continuously and unobtrusively on physiological, behavioral, and activity patterns to help identify subtle digital biomarkers of early neurological changes [34]. This real-time stream consists of gait parameters, sleep patterns, heart rate variability, and social interactions and gives a detailed profile to be monitored longitudinally and identify diseases at an early stage [30]. As an example, digital biomarker applications produce high-resolution data on cognitive and motor processing, voice features, and micro-errors, which may be used to model disease progression and accurately predict conversion events [53]. By combining these passive sensing modalities with multimodal AI strategies, diagnostic sensitivity and specificity will be improved, particularly at the preclinical stages of neurodegenerative disorders [54,55]. Mobile applications such as DailyCog capture data entered by users and response time, to give information about conditions like PD and dementia [36]. State-of-the-art AI models that analyze speech characteristics from smartphone recordings have shown 75% accuracy and an AUC of 0.79 in predicting correlation between acoustic features and cerebrospinal fluid amyloid status, a critical biomarker of early AD [31]. These multidimensional approaches are more valid to the assessment of cognition, functionality, and mobility of patients compared to conventional clinical evaluation, and enhance the diagnosis of the initial stages of neurodegenerative diseases [56] as shown in Figure 1.

## 6. Methodological Evolution, Critical Synthesis, and Open Research Problems

### 6.1. Four Generations of AI in Neurological Diagnostics

#### 6.1.1. Generation 1 (2005–2013): Handcrafted Biomarker Engineering

Handcrafted biomarker engineering approaches, such as Hjorth complexity parameters, Spectral band power, fractal dimensions, and MFCC coefficients, were extracted from EEG, speech, and gait signals and subsequently classified with SVMs or linear discriminant analysis [6,57,58,59]. Sensitivity (70–80%) was achievable when testing on the same recording system used for training, but cross-site validation revealed brittleness. The same feature set lost 12–20% points of accuracy when applied to a different EEG amplifier or room acoustics. This ceiling defined the core lesson: domain-specific feature engineering encodes hardware-dependent assumptions that fail to generalize across heterogeneous acquisition environments [59,60].

#### 6.1.2. Generation 2 (2013–2018): Convolutional Representation Learning

Hierarchical spatial feature learning worked well for transfer across subjects in the same scanner for 1D and 2D CNNs on raw EEG, MRI, or spectrograms, with gains of 5–8% in F1-scores over handcrafted baselines on the CHB-MIT seizure corpus [57,61,62]. For Alzheimer’s disease staging, 3D CNNs trained on ADNI structural MRI achieved AUC values of 0.87–0.92 in predicting the rate of progression from MCI to AD, which is significantly higher than the volumetric baselines [19,63]. The lesson is that deep feature learning not only saved the engineering effort but also boosted performance by 4–10%. However, each clinical cohort is not balanced (4:1 healthy-to-case ratio), and the scanner varies across ADNI sites. Each of the missing longitudinal timepoints were a detriment to the performance, highlighting differences between curated benchmark and real clinical data [64,65].

The unimodal results have an underappreciated implication for the multimodal fusion paradigm used in the following literature. It has been assumed that staging AD would require the integration of structural MRI with other complementary measures such as PET amyloid burden, CSF biomarkers, or neuropsychological scores. However, the 0.87–0.92 AUC scores obtained with 3D CNNs using only structural MRI already approach the ceiling that multimodal fusion was expected to raise. One study quantified this directly, reporting only a 3.4% marginal AUC gain when a full multimodal fusion model was benchmarked against a strong 3D MRI-only baseline [59,61,64]. The gain is real but narrow and must be weighed against the practical cost of multimodal acquisition. PET imaging is unavailable in most memory clinics. CSF collection is invasive, and missing any single modality at inference time requires imputation strategies that themselves introduce variance.

#### 6.1.3. Generation 3 (2018–2023): Attention, Temporal Modelling, and Graph Representations

Bidirectional LSTMs and temporal CNNs were shown to model relationships across multi-minute EEG recordings for better seizure prediction, with LSTM models attaining a maximum of 99.01% sensitivity in the controlled 10-fold cross-validation [12,66]. Graph convolutional networks reframed brain connectivity as a learnable topology. Edges encoded phase-locking value or Granger causality between EEG channels. Spectral GCNs outperformed channel-independent CNNs by 8–11% F1-score on the Temple University Hospital corpus [67,68,69]. Transformer self-attention generalized this further, capturing non-local temporal dependencies without recurrence limitations [70,71]. The lesson is that inductive bias toward the graph structure of the brain leads to better performance. However, interestingly, the GNN results are very sensitive to the functional connectivity metric employed (PLV; when substituted with coherence, results changed by up to 7% in classification accuracy). This introduces an implicit methodological bias that stops the possibility of a meta-analytic comparison [72,73]. Recent reviews document the momentum and remaining gaps in this paradigm, provide a mapping of the current space of EEG decoding foundation models, highlight generalization across montage and pathology as the central open challenge, and assess the necessity for domain-informed architectural priors when scaling up EEG decoding [74,75].

While the third generation established deep learning as the dominant paradigm for automated feature learning from complex neurological data, recent advances in large-scale pre-trained models and foundation model architectures have begun to redefine AI applications beyond conventional task-specific deep learning. These emerging developments form the basis of the fourth generation of AI discussed below.

#### 6.1.4. Generation 4 (2023–Present): Foundation Models and Few-Shot Clinical Generalization

Self-supervised pre-training on large unlabeled corpora, including thousands of structural MRI scans, hours of multichannel EEG recordings, and millions of clinical notes, has started to make it possible to adapt few-shot tasks. BrainBERT-style masked autoencoding on intracranial EEG enables few-shot adaptation for seizure classification [54,76,77]. Vision language models fine-tuned on MRI report pairs enable zero-shot neuroanatomical analysis in low-resource settings [78]. The NeuroMoE mixture-of-experts framework dynamically routes multimodal inputs through specialized expert networks for multi-class neurological disorder classification [7].

These developments require careful consideration of the scale of the pre-training data used, especially for EEG-based models. Whereas BrainBERT-style approaches operated on intracranial recordings numbering in the hundreds of hours, the current frontier of self-supervised EEG representation learning has expanded by orders of magnitude: state-of-the-art transformer-based foundation models are now pre-trained on tens of thousands of hours of heterogeneous EEG data drawn from multiple institutions and recording paradigms [74,75]. The increasing availability of large, diverse pre-training datasets may improve the robustness and generalizability of foundation models; however, further multicenter validation is required before these approaches can be translated into routine clinical practice.

Yet, there is no foundation model for neurology that is validated in a prospective randomized trial with clinical endpoints. In addition, regulatory frameworks that would govern adaptive AI systems are not clearly defined in either the FDA or EMA jurisdiction [63,79,80,81,82,83]. Despite these advances, foundation models remain largely investigational in neurology. Prospective multicenter validation, standardized reporting, regulatory evaluation, and demonstration of clinically meaningful patient outcomes remain necessary before these models can be considered for routine clinical implementation.

### 6.2. Critical Comparative Performance Analysis Across Modalities and Diseases

A systematic review of 47 deep learning studies on AD classification published in 2018–2024 has shown a constant upward trend. Single-site training of the ADNI cohort (n = 1000–2000) reported an AUC > 0.90, whereas independent testing on NACC, OASIS-3, and UK Biobank yields 0.78–0.85 [63,84,85,86]. The 8–18% decrease can be explained by scanner manufacturer differences (1.5 T versus 3 T field strength shifts the cortical thickness by 4–6%), demographic mismatch (the majority of the ADNI population is White, educated, and North American), and acquisition protocol differences. This gap is not addressed by data augmentation or dropout regularization; study designs that will occur in the future at multiple sites are the primary standard of validation required [63,72,84]. Another parallel problem with EEG seizure detection is that the reported accuracy of 99% when using CHB-MIT or Bonn datasets is based on leave-one-segment-out cross-validation, which introduces temporal autocorrelation. The cross-validation obtained when using patient-independent data shows a range of accuracy from 78 to 88%, which is closer to clinical usability [57,62,87].

Performance ceilings are modality specific rather than architecture specific. For PD, wearable accelerometry outperforms the clinical rating scales in test–retest reliability, with an ICC of 0.91 compared to 0.74 for clinical scales. However, it has lower prodromal sensitivity values of 64–72% compared to 84% for the AI model based on ECG and trained on the UK Biobank, which has been able to identify pre-motor PD up to 7 years in advance [2,4,88]. In the case of epilepsy, deep learning on scalp EEG has obtained higher than 95% sensitivity for seizure detection in controlled settings, while ambulatory deployment has resulted in false positive rates of 2–5/hour due to movement artefacts or other sources. This makes side-by-side alerts impractical without additional stages of post-processing [14,15,61,62]. Speech NLP gives MCI a prediction horizon of 6 years with 78.5% accuracy [8]. However, it has a lower sensitivity of 81.1% (meaning that 19% of converters are missed) and is therefore ideal for population screening and not clinical guidance. The need for a multi-objective optimization methodology is highlighted by this modality performance landscape, where the deployment of the device is not simply about improving the accuracy of the metric but involves balancing sensitivity, specificity, cost, invasiveness, and scalability [89,90].

### 6.3. Unresolved Technicaand Clinical Research Problems

#### 6.3.1. Open Problem 1-Prospective Digital Biomarker Validation

Most of the published studies related to AI biomarkers are convenience cohorts of retrospective data, highlighting the need for prospective validation in clinically representative populations. Prospective validation requires pre-specified primary endpoints, standardized biomarker acquisition, appropriate subgroup analyses, and clinically meaningful longitudinal outcomes to establish the clinical validity and utility of digital [79,81,82,91]. For prodromal AD, longitudinal studies involving biomarker-defined at-risk populations and clinically meaningful cognitive outcomes could provide stronger evidence for the clinical utility of AI-derived digital biomarkers. Methodological considerations for clinical trials in prodromal AD and Parkinson’s disease (PD) further emphasize the importance of appropriate trial design and outcome selection [31,48,82].

For prodromal PD, longitudinal digital-biomarker studies incorporating wearable and remote measures may provide complementary information for disease characterization and monitoring. Digital biomarkers have increasingly been investigated for precision diagnosis and longitudinal monitoring of PD, while clinical-trial-informed frameworks emphasize the need for rigorous evaluation before translation into routine clinical practice [51,79]. Methodological studies across AI-based neurological applications further demonstrate the importance of rigorous model development, feature selection, performance evaluation, and independent assessment when interpreting diagnostic accuracy [4,92,93]. These methodological considerations are relevant when evaluating AI-derived biomarkers because high performance in a single dataset does not necessarily establish generalizability or clinical utility.

The clinical translation of AI-based biomarkers also requires consideration of digital-measure development, clinically meaningful change, implementation, and regulatory requirements [79,81,82,85]. Retrospective diagnostic performance alone should therefore not be interpreted as sufficient evidence of clinical utility or readiness for routine clinical implementation. Instead, prospective evaluation in representative populations, standardized outcome assessment, and demonstration of clinically meaningful benefit are important components of the evidence-development pathway [79,81,82].

A closely related and frequently under-reported validation issue is the granularity at which a dataset is split into training and test sets. Many of the studies discussed in this review are derived from multiple overlapping windows, trials, or recording segments from each participant (for example, successive gait cycles, speech utterances, or EEG epochs). if individual segments are randomly divided between training and test sets. When data from the same participant are present in both sets, subject-specific physiological, behavioral, and recording characteristics may be learned by the model, resulting in information leakage and inflated estimates of generalization performance. In contrast, participant-level or subject-independent splitting confines all observations from a given participant to either the training or test set and provides a more stringent assessment of model generalizability. Therefore, performance estimates from studies that do not explicitly report participant-independent data splitting should be interpreted with caution, particularly when comparing results across heterogeneous datasets and studies [92,93]. We flag it here as a methodological caveat that applies across Table 2 and Table 3 and recommend that readers treat performance figures from studies that do not explicitly report patient-independent (subject-level) splitting with additional caution.

#### 6.3.2. Open Problem 2—Demographic Generalizability and Algorithmic Fairness

A systematic analysis of 62 EEG-AI studies found that fewer than 12% reported participant ethnicity and fewer than 5% performed subgroup accuracy analysis by age, sex, or education [80,94]. This is clinically consequential. EEG spectral power varies by age and sex, speech biomarkers differ by native language, and gait parameters vary with height and musculoskeletal comorbidities. Models trained without demographic stratification produce differential diagnostic error rates of 15–25% across subgroups, systematically encoding access disparities into the AI pipeline [65,94]. Solutions require mandatory disaggregated performance reporting (analogous to the FDA’s action plan for AI/ML-based software as a medical device) and deliberate inclusion of underrepresented groups in prospective training cohorts.

#### 6.3.3. Open Problem 3—From Phenomenological to Mechanistic Biomarkers

Existing digital biomarkers detect observable deviations in behavior correlated with disease stage but cannot distinguish amyloid-driven cognitive decline from vascular or TDP-43 pathology at the individual level [95,96]. Integrating digital phenotyping with plasma biomarkers (p-tau217, NfL, GFAP) within unified multimodal AI frameworks would anchor phenomenological signals to mechanistic hypotheses. This is technically challenging: plasma markers and digital assessments operate on different timescales (weeks versus seconds), have incompatible noise structures, and require separate preprocessing before joint modelling [27,97,98]. Architectures that handle asynchronous, heterogeneous time-series data without requiring simultaneous measurement represent an active unsolved research problem [23].

#### 6.3.4. Open Problem 4-Human-AI Collaboration and Clinical Deployment Science

The translation bottleneck is not algorithmic performance alone; it also involves workflow integration and clinician behavior change. Prospective deployment studies of AI diagnostic tools in clinical settings consistently find physician override rates above 70%, alert fatigue from false positives, and performance degradation when applied to unselected ward populations rather than curated research cohorts [80,81,99]. The AI neurology literature lacks frameworks equivalent to radiology AI reader studies or structured usability protocols in surgical robotics. Validated human-AI collaboration models-specifying pre-defined thresholds for acting upon AI output, ordering confirmatory tests, or overriding recommendations-are a prerequisite for safe real-world deployment that remains largely unaddressed in current research programs [89].

## 7. Machine Learning Methodologies for Digital Biomarker Extraction and Analysis

State-of-the-art machine learning models, including supervised, unsupervised, and deep learning models, are at the core of producing actionable digital biomarkers clinically based on multimodal data [100]. These techniques can include various architectures, such as support vector machines, random forests, and gradient-boosted trees for supervised classification; clustering and dimensionality reduction for unsupervised pattern discovery; and deep learning with convolutional or recurrent neural networks applied to spatiotemporal data from neuroimaging, electrophysiology, speech assessments, gait detectors, and wearables [24]. Moreover, longitudinal modelling and the use of real-world evidence make it possible to conduct continuous monitoring and individualized risk evaluation in natural environments. In the medical conditions of AD, PD, and epilepsy, AI systems have demonstrated strong diagnostic and prognostic performance, often matching or exceeding traditional tests in limited conditions; however, performance varies among studies [8,101].

The field of EEG-based deep learning has progressed from shallow CNN feature extractors to architecturally complex pipelines that have directly measurable improvement in performance. Temporal convolutional networks (TCN) provide an improvement of 6–9% in the seizure detection sensitivity on the CHB-MIT benchmark compared with hand-crafted feature pipelines, and the combination of convolutional networks and long short-term memory (CNN-BiLSTM) models combines the advantages of both local waveform morphology and long-range temporal dependency, achieving the best results of 97.8%/98.3% in seizure detection sensitivity/specificity on the BONN epilepsy dataset [57,61]. Additionally, electrode-wise attention mechanisms further enhance electrode-specific discrimination, lowering FP rates up to 34% compared to global pooling architectures [102]. Most importantly, generalization across datasets is still not complete: a model trained on adult scalp EEG exhibits a 12–23% drop in accuracy in pediatric intracranial EEG, highlighting the need for domain-adaptive pre-training strategies [103]. Transformer architectures have transformed beyond NLP in the field of neuroimaging analysis. Applying vision transformers to 3D structural MRI data obtains 0.92–0.95 AUC for AD classification, with spatial interpretability of the attention maps in the form of localized hippocampal and entorhinal atrophy without prior anatomical knowledge [70,104]. The cross-modal transformer with dual-stream encoders for paired MRI-PET inputs achieves an improvement of 4.3% compared to the late-fusion baseline methods in multi-class AD staging and shows good performance in the 30% missing modality scenario [105]. The NeuroMoE mixture-of-experts framework enables modality-specific routing, enabling transformer experts to specialize on both EEG and MRI, as well as on clinical features, without incurring a significant increase in parameters compared to a dense multi-task network, achieving a 40% parameter reduction [7]. Furthermore, graph neural networks (GNNs) provide a principled solution to model brain topology. Dynamic graph convolutional networks (dGCNs) based on EEG functional connectivity matrices are able to model the propagation dynamics of seizures that are not captured by channel-independent models, with F1-score gains ranging from 8 to 11% on the Temple University Hospital EEG corpus [67,68]. The use of Brain GNN for fMRI connectomes introduces interpretable node selection mechanisms that identify regions of the brain relevant to the disease, while simultaneously providing improved performance and clinical explainability [76,77]. These spatiotemporal modelling techniques significantly increase the scope of clinical neurological tools available to AI by modelling complex neural dynamics that are not modelled by simpler methods [15].

Additionally, large corpora of neuroimaging data serve as a foundation for pre-training transfer learning models to boost downstream diagnostic performance. Brain BERT-style masked autoencoder pre-training on EEG signals reduces a seizure classifier’s need for a labelled dataset by 60% and allows for effective fine-tuning with as little as 200 labeled epochs [106]. This paradigm is particularly effective when only little information exists about rare disease subtypes. External validation using independent cohorts shows that published single-site accuracy estimates are systematically overoptimistic by 8–18% compared to multi-site replication results [6,86]. This highlights the need for prospective validation protocols, preregistered analysis plans, and diversity-stratified performance reporting. In addition to the diversity arising from the various types of data sources (wearables, smartphones, imaging, etc.), data quality issues, labelling errors, and non-uniform or missing preprocessing pipelines are challenges that must be overcome to ensure the robustness of trained models [1,99,107,108]. However, the demonstrated efficacy of reduced model performance across various demographics, scanner protocols, or ethnic groups has led to the need for federated learning, domain adaptation, and multicenter dataset efforts to improve generalizability [1,3,4]. The main obstacles in translation are workflow integration, resistance to adoption, and regulatory uncertainty, which is enhanced by the risk of bias, healthcare access disparities, and ad hoc implementation systems that require comprehensive data curation, interpretable designs, and custom implementation structures [79,80]. These frameworks are important to help fill the gap between the potential of the research findings and their possible application in a variety of clinical environments to provide equal opportunities and successfully implement AI-driven diagnostic tools. In fact, the integration of various data streams, such as neuroimaging, physiological, and behavioral data, using multimodality is consistently better than single-modality algorithms, where deep learning is better at recognizing patterns and traditional machine learning provides a more understandable one [109]. Genomics, imaging, and EHR data are especially suited to a multimodal AI framework, which holds great promise for precision neurology [25]. Proteomics-based multi-omics integration is also becoming a powerful tool to complement digital phenotyping, and proteomics has been reported as the most studied omics layer when investigating AD. In addition, the integration of proteomics with transcriptomics is often reported to reveal molecular pathways associated with disease [98].

**Figure 2:**
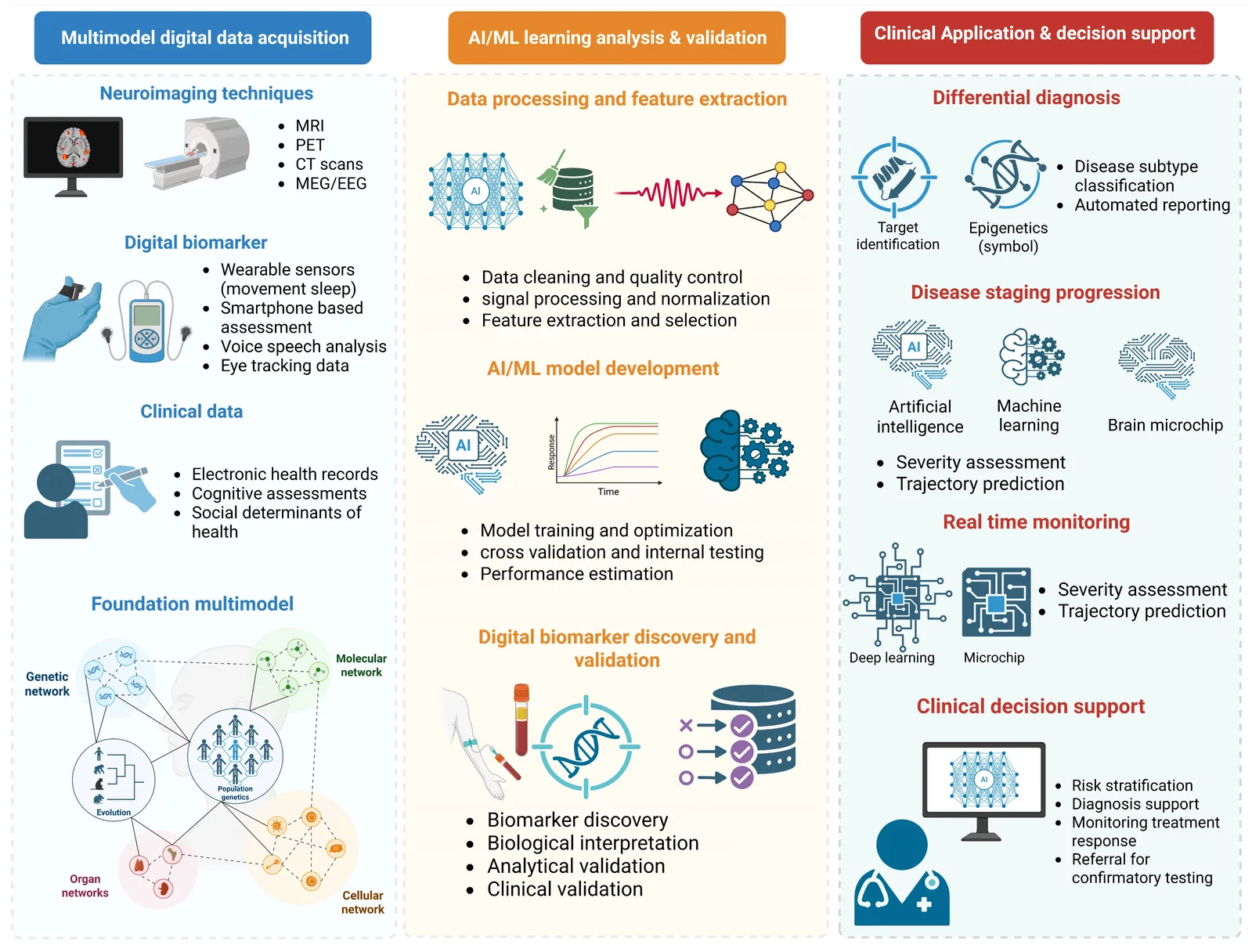
**Overview of the AI-enabled workflow for the development and clinical application of multimodal digital biomarkers in neurological disorders.** The workflow consists of three sequential stages. (1) Multimodal digital data acquisition: collection of data from neuroimaging modalities (MRI, PET, CT, MEG/EEG), digitally acquired biomarkers (wearable sensors, smartphone-based assessments, voice and speech analysis, and eye tracking), clinical data (electronic health records, cognitive assessments, and social determinants of health), and multimodal data integration. (2) AI/ML learning, analysis, and validation: preprocessing of multimodal data using quality control, signal processing, normalization, and feature extraction, followed by machine learning and deep learning model development, performance evaluation, and digital biomarker discovery with analytical and clinical validation. (3) Clinical application and decision support: validated AI-enabled digital biomarkers support differential diagnosis, disease staging and progression assessment, real-time disease monitoring, and clinical decision support, including risk stratification, diagnostic support, monitoring of treatment response, and referral for confirmatory clinical evaluation. The figure summarizes the end-to-end AI workflow from multimodal data acquisition to clinically relevant decision support and highlights the importance of biomarker validation before clinical implementation. The figure was created in https://BioRender.com.

AI-enabled digital biomarker development integrates multimodal data, including neuroimaging, digital physiological and behavioral signals, and clinical information with advanced machine-learning and deep-learning methods. Following preprocessing, feature extraction, model development, and validation, these approaches support biomarker discovery and translation into clinical applications such as differential diagnosis, disease monitoring, and clinical decision support (Figure 2).

## 8. Translational and Clinical Integration Challenges

Although these applications are expected to have positive benefits for neurodegenerative disease diagnostics, several challenges for translation into clinical use are present, including issues in machine learning and digital biomarker development [1,3,110]. Validation is still not well developed, and models tend to be less generalized to different cohorts, institutions, devices, and demographics because of the variability in data and overfitting [1,4,99]. Black box AI decisions, workflow distortions, regulatory ambiguities, and risks of bias that contribute to healthcare inequities undermine clinician confidence [3,10]. Key technical, clinical, ethical, and societal challenges affecting the translation of AI-based neurological diagnostics are summarized in Table 4.

### 8.1. Data Heterogeneity and Generalizability Across Clinical Settings

The heterogeneity of datasets based on diverse sources (wearables, smartphones, imaging, etc.) is both difficult and comes with several challenges, including the inconsistency of data quality, labeling errors, and lack of standardization. These challenges undermine the robustness of a model [1,98,106,107]. Research indicates reduced effectiveness in the application of the models to various groups of people, scanners, or ethnicities, which requires federated learning, domain adaptation, and multicenter datasets to enhance the generalizability of results [1,3,4].

### 8.2. Model Interpretability and Clinician Trust

Deep learning models are considered a barrier to clinician trust and adoption because black box-based predictions do not allow interpreting the reasons behind making decisions [3,5,9]. To address this gap, explainable AI methods, including gradient activations and models that are inherently interpretable, are needed, as well as rigorous validation systems that achieve reproducibility and fairness [3,5,99]. Recent research showed the usefulness of XAI frameworks, such as SHAP and LIME, for Parkinson’s disease diagnosis, where an integrated XAI framework with feature engineering resulted in an accuracy of 92%, balanced sensitivity, and high interpretability [29,113]. The multimodal explainable frameworks that integrate explainable vision transformers with attention heatmaps and integrated gradients on the PPMI database have been further deepening the understanding of doctors on deep learning decision making in the field of PD diagnosis [113,114].

### 8.3. Integration into Clinical Workflows and Adoption Barriers

Smooth workflow integration is hampered by barriers such as high computational load, software engineering requirements, and opposition of overworked clinicians [1,3,99]. It is suggested to implement sandbox testing; stakeholder co-creation among patients, providers, and policymakers; and a user-friendly design that can enable quick deployment and close the digital literacy divide [3,10].

### 8.4. Bias, Healthcare Equity, and Global Health Implications

AI models have the potential to reproduce biases from nonrepresentative training data, worsening inequities in low-resource environments and underserved populations [1,3,24]. The digital divide represents an access, infrastructure, and literacy divide that requires equity-centered data curation, a non-discriminatory cohort, and light, offline-capable tools to achieve global scalability [3,4,5]. Moreover, a lack of standard data capture and labelling practices among healthcare facilities is a major obstacle to the creation of powerful and generalized AI models [111,112].

## 9. Ethical, Privacy, and Regulatory Considerations

Combining AI-based digital biomarkers and machine learning models to detect neurodegenerative diseases early presents some of the most significant ethical, privacy, and regulatory issues that must be actively addressed to ensure the promotion of trust, equity, and safe clinical implementation [1,3,5,24]. These issues include informed consent to use sensitive multimodal data, algorithmic transparency to address the risks of black box opaqueness, the risk of bias continuing to perpetuate healthcare disparities, and the necessity to have effective data governance as the world’s neurological burdens are increasing [3,9,16].

### 9.1. Ethical Considerations and Equity

The ethical demands focus on patient autonomy, cognitive liberty, and equal access, especially with the expansion of AI to various populations up to low- and middle-income nations [1,3,5]. The process of informed consent should clearly specify the dangers and advantages of using data, particularly in the case of patients with cognitive disabilities, with Institutional Review Boards guaranteeing control over algorithmic bias and disparities [9,24]. To overcome the tension among the medical, IT, legal, and ethical realms, interdisciplinary collaboration is needed, facilitating mental privacy protection through inclusive data curation and user-friendly designs [1,3].

### 9.2. Privacy and Data Security

Big data generated by neuroimaging, wearables, and genome studies require strict privacy protection, including encryption, federated learning to process it in a decentralized manner, and compliance with regulatory standards, including the General Data Protection Regulation (GDPR) and the Health Insurance Portability and Accountability Act (HIPAA), to avoid breaches, which may undermine the level of patient trust [3,9,23]. Federated learning (sFL) methods have shown that collaborative model training is feasible in AD detection while avoiding the sharing of raw data and maintaining both GDPR compliance and data utility [115,116]. However, its effectiveness depends on appropriate governance, cybersecurity, validation, and institutional implementation rather than guaranteeing regulatory compliance or eliminating data heterogeneity. Recent studies have demonstrated the feasibility of secure federated learning for Alzheimer’s disease and EEG-based dementia classification while maintaining data utility and patient privacy [112,116].

### 9.3. Regulatory Frameworks and Policy Implications

Regulatory ambiguity continues to hinder the clinical translation of AI-enabled digital health technologies and highlights the need for risk-based governance, strict validation frameworks, medico-legal clarity, and continuous policy re-evaluation in which innovation is balanced with patient safety monitoring [3,10]. A major emerging regulatory challenge concerns the requirement for patient-centered meaningful outcomes in digital biomarkers and digital endpoints, particularly as agencies such as the U.S. Food and Drug Administration increasingly emphasize evidence that these measures reflect clinically relevant benefits in patients’ daily lives. Recent regulatory setbacks involving digital gait and activity endpoints demonstrate that technical accuracy alone is insufficient without proof of real-world clinical utility and patient relevance [80,81]. Consequently, future frameworks should prioritize standardized validation protocols, explainable AI standards, stakeholder co-creation, and robust patient-centered outcome assessment. In parallel, digital health equity guidelines are essential to bridge disparities in access, literacy, and health outcomes across diverse global populations [3,5,24].

Despite considerable progress in AI-enabled digital biomarkers, regulatory pathways remain heterogeneous across jurisdictions and continue to evolve. Future clinical implementation will require standardized validation protocols, transparent reporting, patient-centered outcome measures, post-deployment performance monitoring, and close collaboration among clinicians, regulators, industry, and patients. Frameworks such as TRIPOD+AI provide valuable guidance for reporting AI prediction models and may facilitate future regulatory evaluation and clinical translation.

## 10. Future Directions and Emerging Frontiers

Although substantial progress has been achieved in developing AI-enabled digital biomarkers, several emerging technologies remain investigational and require rigorous multicenter validation before routine clinical implementation. The following sections summarize promising future directions that may enhance the scalability, interpretability, and clinical utility of AI systems in neurological disorders.

### 10.1. Federated Learning and Privacy-Preserving AI Approaches

Beyond its role as a privacy-preserving strategy, federated learning represents an emerging computational framework for multicenter model development that may improve model robustness and generalizability by enabling collaborative learning across geographically distributed institutions. Future research should focus on standardized governance, harmonized data preprocessing, secure model aggregation, and prospective multicenter validation to determine its real-world clinical utility. Multi-institutional federated learning for PD detection based on speech signals in different languages has shown that the federated models are able to outperform all the local models with high diagnostic accuracy while still preserving strict privacy [115]. Another level of security for sensitive neurological data is provided by secure multi-party computation and federated learning [116,117].

### 10.2. Explainable AI for Clinical Decision Support in Neurology

Future explainable AI, which includes gradient activations, inherently interpretable models, and benchmarks, can be of critical importance to clinician trust, disclosing decision rationales in the black box deep learning systems of neurodegenerative diagnostics [3,4,24]. By enhancing reproducibility and bias reduction in the detection of prodromal Parkinson’s disease and Alzheimer’s disease, large-scale validation of XAI in multimodal imaging and biomarker analysis will contribute to adoption [1,10]. XAI frameworks employing SHAP and LIME in the field of PD prediction have shown that XAI can significantly improve the fairness, performance, and interpretability of the model when combined with SMOTE and feature engineering, thereby making it a valuable and interpretable diagnostic tool that can be used in the clinical context to support clinical decision making [113]. Multimodal XAI approaches, which integrate neuroimaging, motor, and non-motor symptom information, succeed in providing interpretable prediction of PD with clear clinical relevance [118].

### 10.3. Integration with Precision Medicine and Digital Therapeutics

**Figure 3:**
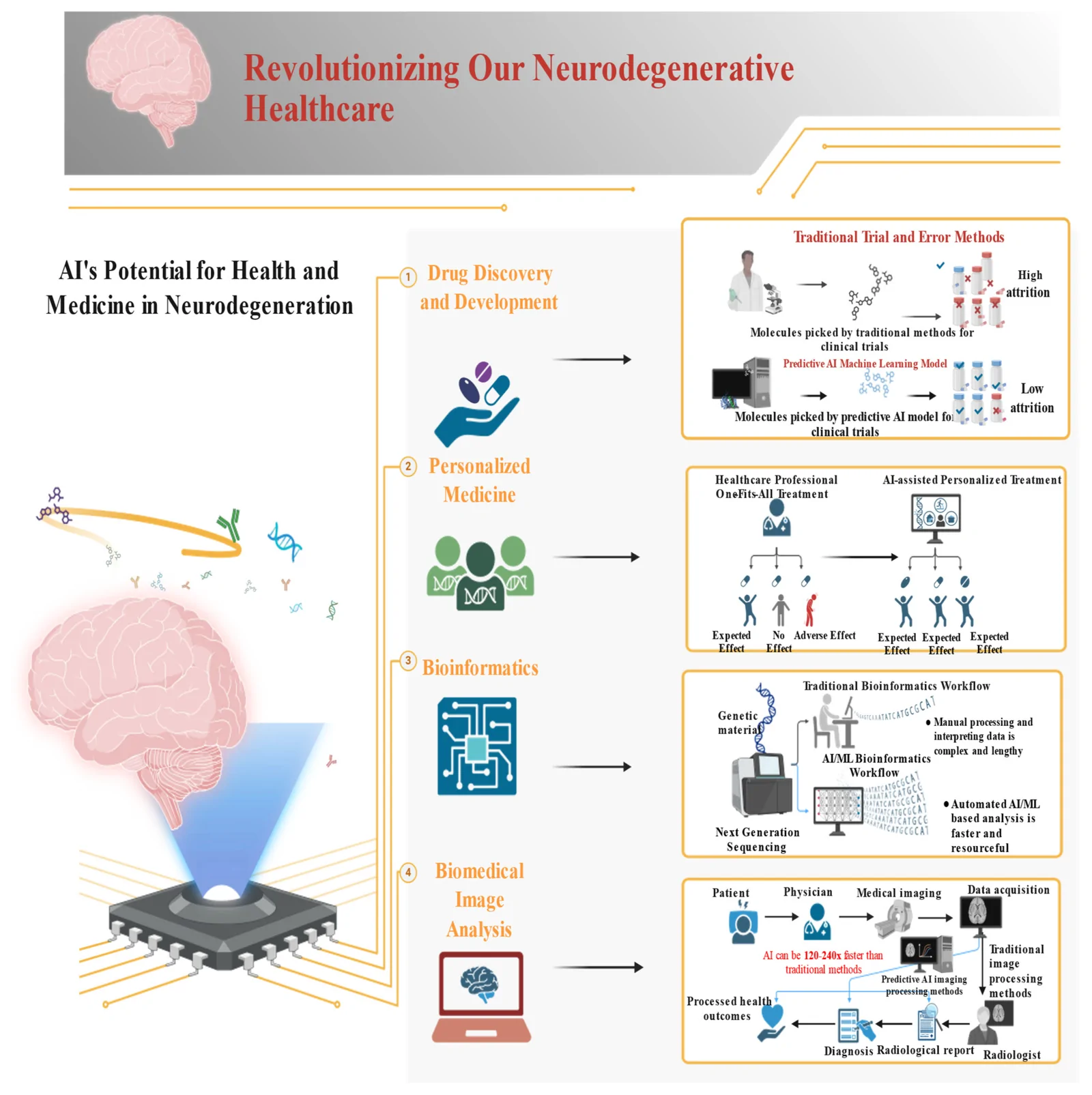
**Schematic overview of representative applications of artificial intelligence (AI) in neurological healthcare.** The figure highlights four major application domains: (1) Drug discovery and development, where AI-assisted virtual screening and predictive modeling facilitate candidate prioritization and optimization compared with conventional trial-and-error approaches. (2) Personalized medicine, where AI integrates multimodal clinical and patient-specific information to support individualized risk assessment and treatment planning. (3) Bioinformatics, where AI accelerates the analysis and interpretation of high-throughput genomic and molecular datasets to identify biologically relevant patterns and potential therapeutic targets. (4) Biomedical image analysis, where AI-based image processing and pattern recognition improve the detection, classification, and interpretation of neuroimaging data. Collectively, these examples illustrate the broad potential of AI to enhance biomedical research and clinical decision making across neurological disorders. The figure represents representative applications of AI and should not be interpreted as a validated or sequential clinical workflow. The figure was created in https://BioRender.com.

Precision medicine is integrated with AI using genomic profiles, digital biomarkers, and prognostic models to deliver personalized interventions, speeding up drug discovery and demonstrating therapeutic predictability using multi-omics and biophysical stimuli [3,24]. Digital therapeutics such as real-time monitoring and telerehabilitation wearables using brain–computer interfaces are more likely to improve neuroplasticity and patient stratification in clinical trials [1,10,30]. Integrating multi-omics approaches, such as proteomics, is moving towards precision medicine for AD, providing insights into molecular pathways and patient stratification beyond clinical phenotypes [98]. The future of precision neurology lies in multimodal AI frameworks that combine genomics, imaging, and electronic health records, facilitating comprehensive disease risk and progression models and a more personalized approach to treatment response [25]. Beyond diagnosis, AI is increasingly transforming the landscape of neurological healthcare. The clinical translation of AI-enabled digital biomarkers involves a sequential process beginning with digital biomarker identification and validation, followed by patient stratification, risk prediction, and AI-assisted clinical decision support. Validated AI outputs may subsequently inform therapeutic decision making within established clinical workflows after appropriate confirmatory assessment. This translational pathway illustrates how AI-enabled digital biomarkers can complement existing diagnostic and therapeutic strategies while emphasizing the importance of rigorous analytical and clinical validation before routine implementation (Figure 3).

### 10.4. Roadmap Towards Real-World Clinical Deployment

Translation of AI-enabled digital biomarkers into routine neurological care will require standardized validation protocols, prospective multicenter studies, regulatory approval pathways, stakeholder engagement, equitable data collection, and integration within existing clinical workflows. The use of digital literacy campaigns to bridge the translational gaps in multidisciplinary collaborations across academia, industry, and regulators will guarantee the use of scalable and validated AI tools in global neurology practices [3,9,24]. This roadmap should clearly bring up how to create extensive, objective, and open datasets, which are the basis of formidable AI work in neurology [3]. Fidelity in the translation of research performance into actual clinical outcomes relies on systematic approaches to real world AI implementation, informed by clinical trials frameworks [80].

## 11. Conclusions

The evidence analyzed in this review indicates that the field is moving from proof-of-concept demonstrations toward increasingly rigorous validation, but most applications remain investigational rather than ready for routine clinical deployment, particularly for screening asymptomatic or prodromal populations. Significant gaps exist when comparing published benchmark performance with real-world validation. Recent advances in transformer-based architecture, graph neural networks, and multimodal learning have expanded the analytical capabilities of AI-enabled digital biomarkers. However, direct comparisons among AI architectures remain challenging because published studies differ substantially in disease definitions, datasets, preprocessing methods, sample characteristics, outcome measures, and validation strategies. Consequently, no single AI architecture can currently be considered universally superior across neurological disorders or digital biomarker modalities. Annotation bottlenecks and better cross-site generalizability are likely to depend on the next generation of foundation models pre-trained on large, neuroimaging corpora. However, academic tools are missing from prospective trial pathways, standardized reporting of performance, and post-market surveillance to meet the distinct, jurisdiction-specific regulatory requirements of FDA clearance/approval and EU CE marking for adaptive AI diagnostics. Overall, the current evidence supports scientific and clinical promise but does not yet establish readiness for population screening or routine implementation within standard clinical workflows. Most applications require prospective, multicenter, patient-independent validation; calibration assessment in clinically representative populations; comparison with standard care; clinical utility and cost-effectiveness analysis; fairness and subgroup performance evaluation; and regulatory review before clinical implementation. Future priorities include prospective multicenter validation, standardized reporting, patient-independent testing, federated learning for privacy-preserving collaboration, lightweight AI architectures suitable for resource-limited settings, transparent model evaluation, and equitable performance assessment across diverse patient populations.

It is also worth stating plainly what would need to follow a positive result from one of the digital biomarker tools discussed in this review since this is rarely addressed in the primary literature itself. A positive or elevated-risk digital biomarker output is best understood as a trigger for confirmatory clinical and, where available, biological assessment (for example, clinical evaluation, neuroimaging, or fluid-biomarker testing) rather than as a diagnosis in itself. This matters clinically because current disease-modifying therapies for Alzheimer’s disease, where they exist, are approved only for selected early symptomatic patients with confirmed amyloid pathology, not for asymptomatic individuals identified solely by a digital screening tool. A positive screen therefore does not, by itself, establish treatment eligibility. It also matters practically because, in a low-prevalence screening setting, even a highly specific test generates a meaningful number of false positives in absolute terms, with associated downstream costs, anxiety, and burden on confirmatory testing pathways that have not been evaluated for most of the tools discussed here. We therefore frame digital biomarkers throughout this review as candidate triage or enrichment tools that may help prioritize individuals for confirmatory testing or closer monitoring, rather than as standalone diagnostic or treatment eligibility instruments. A full cost-effectiveness and psychological impact analysis of this triage pathway was outside the scope of the studies we reviewed and is identified here as a priority for future work.

## Figures and Tables

**Table 1:** Global burden of key neurological disorders.

Disorder	Global Burden/Prevalence	Projected Increase	Ref.
Neurological disorders	443 million disability-adjusted life years (2021), affecting 3.4 billion people	50% increase in DALYs by 2040	[3,16]
PD	~11.8 million people (2021); 3% of individuals over 65, up to 5% over 85	Cases expected to reach ~25.2 million by 2050	[18]
Epilepsy	~50 million people worldwide		[12]
AD	~55 million people globally (dementia, all causes; AD ~60–70%)	Rise to 139 million by 2050	[1,17]

**Table 2:** AI-driven digital biomarker modalities for neurological disorders.

Digital Biomarker Modality	Data Captured/Features	Examples of Application/Features	Ref.
Oculomotor signatures and eye tracking	Saccades, smooth pursuit, fixations, pupil dynamics, blinks, gaze patterns	Early disruptions in PD and AD, differentiation of NDs via CNNs on fixations/saccades	[1]
Speech and voice analysis	Prosody, phonation, speech fluency, rhythm, vocabulary, linguistic patterns, acoustic features (dysphonia/dysarthria)	Prediction of MCI-to-AD progression, early PD detection (iRBD), correlation with amyloid status	[9,30,31]
Facial expressions and motor dynamics	Hypomimia, facial bradykinesia, action units, emotional expressivity, facial mobility, gait, posture	Early PD detection (hypomimia, bradykinesia), quantification of day-to-day symptom variations, differentiation of NDs	[1,11,32]
Cognitive and behavioral digital phenotyping	Memory, executive function, attention, reaction time, daily routines, cognitive-motor independence	Early classification of pre-dementia, MCI, and prodromal stages; prediction of MCI-to-dementia transition	[1,33,34]
Passive sensing	Gait parameters, sleep patterns, heart rate variability, social interactions, motor processing, voice characteristics, micro-errors	Continuous monitoring of early neurological changes, disease progression modeling, precise prediction of conversion events	[11,33,34,35,36]

**Table 3:** Representative studies of AI-enabled digital biomarkers for neurological disorders: clinical applications, digital biomarker modalities, artificial intelligence methodologies, validation strategies, and diagnostic performance.

Disorder	Clinical Task	Target Population/Disease Stage	Digital Biomarker Modality	AI/ML Technique	Cohort/Sample Size	Reference Standard/Comparator	Validation Strategy	Key Performance	Major Limitations	Ref.
PD	Diagnostic classification	Early PD	Eye-tracking data (saccades, pupil, blinks)	CNN/Linear mixed models	Reported in original study	Clinical PD diagnosis	Internal validation	ROC-AUC 0.88; sensitivity 83%; specificity 78%	Single center, internal validation	[1]
Prodromal PD	Population screening	Prodromal PD	Eye tracking (rapid eye movements)	Data mining	Reported in original study	PPMI diagnosis	Internal validation	96% accuracy	Proof of concept	[1,27]
Prodromal PD	Risk prediction	Prodromal PD	Multimodal (ECG/MRI/AI)	AI	Reported in original study	Clinical diagnosis	Internal validation	Up to 84% sensitivity	Small cohort	[2,4]
Prodromal PD	Risk prediction	At-risk individuals	Wearable accelerometry	ML	Reported in original study	Clinical follow-up	Internal validation	Outperforms genetic and lifestyle models in accuracy, sensitivity, specificity	No external validation	[4]
PD	Diagnostic classification	Established PD	Wearables gait sensors	Deep learning	Reported in original study	MDS clinical diagnosis	Internal validation	>90% accuracy	Retrospective	[11,37]
PD	Symptom monitoring	Established PD	Smartphone accelerometer and gyroscope	AI/ML	Reported in original study	UPDRS motor assessment	Internal validation	High precision in quantifying bradykinesia and gait impairment	Limited clinical validation	[10,30,37]
PD (hypomimia)	Diagnostic classification	Early PD	Facial video	Deep learning	Reported in original study	Clinical assessment	Internal validation	85% accuracy	Limited dataset	[1,38,39]
Prodromal PD	Screening	iRBD patients	Voice	AI analysis	Reported in original study	Clinical diagnosis	Internal validation	Balanced accuracy 89% (males), 70% (females)	Sex-specific performance	[4,30]
Prodromal PD	Screening	iRBD patients	voice	CNN	Reported in original study	Clinical diagnosis	Internal validation	Accuracy 90.45%	Single cohort	[30]
MCI to AD	Conversion prediction	Mild Cognitive Impairment	Speech+ demographics	NLP	Reported in original study	Clinical conversion	Internal validation	Accuracy 78.5% and sensitivity 81.1%	No external validation	[9]
Epilepsy	Seizure prediction	Established epilepsy	EEG	ML	Multiple cohorts	Clinical seizure onset	Mixed validation	Advances in seizure prediction	Highly heterogeneous datasets	[6,12]
Sleep apnea	Screening	Adults	Wearables	AI	Meta-analysis	PSG	External synthesis	Pooled accuracy of 0.893	Study heterogeneity	[8]
Pre-dementia	Risk prediction	Cognitively normal	BIOCARD	Granular computing	BIOCARD cohort	Clinical diagnosis	Internal validation	Prediction of pre-dementia stages	Cohort-specific	[1]
MCI	Diagnostic classification	MCI	Web-based testing (wordlist, reaction, trail-making)	ML classifiers	Reported in original study	Clinical diagnosis	Internal validation	ROC-AUC 0.71–0.84	No external validation	[1]
Dementia	Diagnostic classification	Dementia	Web-based testing (wordlist, reaction, trail-making)	ML classifiers	Reported in original study	Clinical diagnosis	Internal validation	ROC-AUC 0.86–0.94	Internal validation only	[1]
MCI-to-Dementia	Conversion prediction	MCI	Altoida app data	ML	Reported in original study	Clinical conversion	Internal validation	AUC 0.92 prediction	Needs prospective validation	[1,35]
Early AD	Amyloid prediction	Early symptomatic AD	Smartphone	AI models	Reported in original study	CSF amyloid	Internal validation	75% accuracy and AUC 0.79	Biomarker correlation study	[9,31]

AD: Alzheimer’s disease; AI: artificial intelligence; AUC: area under the receiver operating characteristic curve; CNN: convolutional neural network; CSF: cerebrospinal fluid; ECG: electrocardiography; EEG: electroencephalography; iRBD: isolated rapid eye movement sleep behavior disorder; MCI: mild cognitive impairment; ML: machine learning; MRI: magnetic resonance imaging; NLP: natural language processing; PD: Parkinson’s disease; PPMI: Parkinson’s Progression Markers Initiative; ROC: receiver operating characteristic; UPDRS: Unified Parkinson’s Disease Rating Scale.

**Table 4:** Challenges and limitations in AI/ML integration for neurological diagnosis.

Category	Specific Challenge	Impact/Description	Ref
Data challenges	Data heterogeneity and variability	Inconsistencies in data quality, labeling errors, lack of standardization across diverse sources (wearables, imaging, etc.)	[1,99,107]
	Limited generalizability	Diminished model performance across different populations, institutions, devices, ethnicities due to unrepresentative training data and overfitting	[1,3,4]
Model and technical challenges	Model interpretability (black box decisions)	Opacity for deep learning models hinders clinician trust, limits understanding of decision rationales	[3,5,10]
	High computational demands	Significant computational resources and software engineering needed for integration	[1,3,99]
Clinical integration	Clinician confidence and adoption	Lack of trust in AI decisions, workflow disruptions, resistance from overburdened clinicians	[3,10]
	Regulatory uncertainties	Ambiguities in regulatory frameworks hinder translation and widespread adoption	[3,10]
Ethical and societal challenges	Bias and healthcare inequities	AI models risk perpetuating biases from unrepresentative data, exacerbating disparities, especially in low-resource settings	[1,3,24]
	Digital divide	Disparities in access, infrastructure, and digital literacy impact equitable deployment and outcomes	[3,4,5]
Practical hurdles	Lack of standardized protocols	Absence of common data capture and labeling practices impedes robust and generalizable AI model development	[111,112]

## Data Availability

No new data were created or analyzed in this study. Data sharing is not applicable to this article.
